# Use of PRP, PRF and CGF in Periodontal Regeneration and Facial Rejuvenation—A Narrative Review

**DOI:** 10.3390/biology10040317

**Published:** 2021-04-10

**Authors:** Eitan Mijiritsky, Haya Drora Assaf, Oren Peleg, Maayan Shacham, Loredana Cerroni, Luca Mangani

**Affiliations:** 1 Department of Otolaryngology, Head and Neck and Maxillofacial Surgery, Tel-Aviv Sourasky Medical Center, Sackler Faculty of Medicine, Tel Aviv 6139001, Israel; mijiritsky@bezeqint.net (E.M.); orenpeleg@gmail.com (O.P.); 2 The Maurice and Gabriela Goldschleger School of Dental Medicine, Tel Aviv University, Tel Aviv 6997801, Israel; 3 Faculty of Dental Medicine, Hebrew University of Jerusalem, Jerusalem 9190401, Israel; Haya.assaf@mail.huji.ac.il; 4 School of Social Work, Ariel University, Ariel 40700, Israel; 5 Department of Translational Medicine and Clinical Science, University of Tor Vergata, 00133 Rome, Italy; cerroni@uniroma2.it (L.C.); manganiluca@yahoo.it (L.M.)

**Keywords:** PRP, PRF, CGF, facial rejuvenation, autologous platelet concentrates, periodontal regeneration

## Abstract

**Simple Summary:**

Growth factors play a vital role in cell proliferation, migration, differentiation and angiogenesis. Autologous platelet concentrates which contain high levels of growth factors are used in many fields of dentistry. The current review is designed to provide clinical information regarding the use of three autologous platelet concentrates techniques in periodontal regeneration and facial rejuvenation: platelet-rich plasma, platelet-rich fibrin and concentrated growth factor techniques. The aim is to provide the clinician with an up-to-date overview of autologous platelet concentrates evolution over the past decade, clinical indications for use and advantages and limitations of each technique. This article was written in clinical orientation and is designed to provide clinicians with reliable and useful information applicable to their clinical work. Overall, platelet-rich plasma is mainly used in cases of hard and soft tissue procedures, while platelet-rich fibrin is used in gingival recession and treatment of furcation and intrabony defects; concentrated growth factor is mainly used in bone regeneration. In the field of facial rejuvenation, the use of platelet-rich plasma promotes tissue remodeling in aged skin and may be used as an adjuvant treatment to lasers; platelet-rich fibrin holds significant potential for stimulated dermal augmentation, and concentrated growth factor treatment could improve the survival and quality of fat grafts.

**Abstract:**

Growth factors (GFs) play a vital role in cell proliferation, migration, differentiation and angiogenesis. Autologous platelet concentrates (APCs) which contain high levels of GFs make them especially suitable for periodontal regeneration and facial rejuvenation. The main generations of APCs presented are platelet-rich plasma (PRP), platelet-rich fibrin (PRF) and concentrated growth factor (CGF) techniques. The purpose of this review is to provide the clinician with an overview of APCs’ evolution over the past decade in order to give reliable and useful information to be used in clinical work. This review summarizes the most interesting and novel articles published between 1997 and 2020. Electronic and manual searches were conducted in the following databases: Pubmed, Scopus, Cochrane Library and Embase. The following keywords were used: growth factors, VEGF, TGF-b1, PRP, PRF, CGF and periodontal regeneration and/or facial rejuvenation. A total of 73 articles were finally included. The review then addresses the uses of the three different techniques in the two disciplines, as well as the advantages and limitations of each technique. Overall, PRP is mainly used in cases of hard and soft tissue procedures, while PRF is used in gingival recession and the treatment of furcation and intrabony defects; CGF is mainly used in bone regeneration.

## 1. Introduction

The use of platelets for regenerative medicine has increased in recent years. Platelets, which contain growth factors, play major roles in cell migration, proliferation, differentiation and angiogenesis and are associated with the tissue regeneration process. Autologous platelet concentrates (APCs) are produced by the centrifugation of venous blood at different speeds and the use or non-use of thrombin and anticoagulant. As a result of these processing protocols, a fibrin clot is formed that contains platelets and leukocytes [1]. The main generations of APCs are platelet-rich plasma (PRP), platelet-rich fibrin (PRF) and concentrated growth factor (CGF). The efficacy of platelet concentrates in promoting wound healing and tissue regeneration has been at the center of scientific interest over the past few decades. Platelets include growth factors (GFs) such as basic fibroblast growth factor (bFGF), vascular endothelial growth factor (VEGF), insulin-like growth factor-1 (IGF-1), transforming growth factor β-1 (TGF-β1) and platelet-derived growth factor-BB (PDGF-BB). There are significant differences in the amounts of GFs produced using the three different APC techniques (CGF, PRF and PRP). PRF and CGF produce significantly more GFs during the procedure as compared to PRP. The levels of bFGF in CGF and PRF are significantly higher than in PRP. However, the levels of the other growth factors abovementioned do not differ significantly among the different APCs [2]. The current narrative review sought to address the increasing usage of APCs in the dentistry, with special attention given to periodontal regeneration and facial rejuvenation, with the latter receiving increasing attention from dentists worldwide [3].

There is also a mechanical impact of biomaterials on cell regeneration, repair and homeostasis. As previously described by Kraft et al., dental pulp stem cells (DPSCs) have the potential to repair and generate teeth and bone [4]. DPSCs can differentiate to odontoblastic-like cells and enable regeneration. Odontoblasts and DPSCs serve as key regulators for dentin formation and its repair due to their ability to sense fluid shear stress, such as the biomechanical forces that occur due to trauma over the cell surface [5,6].

The term “dentinogenesis” refers to the complex dentin pulp which responds to external mechanical stress and repairs itself by forming dentin [7]. The pulp senses the external physical and chemical changes by a signal transduction pathway and then differentiates and forms odontoblastic-like cells, which enable regeneration [8].

Furthermore, stem cells in the oral cavity express mesenchymal cell-like features and are attracting interest due to the fact that they can be easily isolated from teeth extracted due to irreversible periodontitis or for orthodontic reasons and also because of the large amount of cells that can be produced [9,10]. Different kinds of stem cells in the oral cavity can differentiate to different lineages of cells [11]. For example, gingival mesenchymal stem cells can easily differentiate and proliferate, even better than bone marrow stem cells [6,12]. Periodontal ligament stem cells (PDLSCs) can differentiate into adipocytes, collagen-forming cells and cementoblast-like cells. After transplantation, these cells have shown an ability to generate a cementum/PDL-like structure and enable the repair of the periodontal tissue. Hence, these cells have the potential to repair tissues destroyed by periodontal diseases [13]. Stem cells from human exfoliated deciduous teeth have the ability to differentiate into a variety of cell types, such as adipocytes, odobtoblasts and neural cells. These cells have the ability to generate dentin and induce bone formation [14]. From the above, it can be concluded that there are various mechanisms that allow the tissues to maintain homeostasis and regeneration, including biomaterials, mechanical forces exerted on the tooth that stimulate tooth repair, GFs and key signaling pathways.

The current review will discuss the main APCs: PRP, PRF and CGF. PRP, which was first described by Whitmen et al., is prepared by the centrifugation of autologous whole blood together with thrombin and calcium chloride, to form a “platelet gel” [15]. The second generation of APCs, PRF, was developed by Choukroun and described first by Dohan et al. The preparation of PRF does not require the addition of any exogenous material [16]. The newest APC, CGF, was first defined by Sacco. CGF is produced in a manner similar to that used to produce PRF but involves different centrifugation speeds [17]. In the current review, the use of these three techniques in periodontal regeneration and facial rejuvenation will be discussed, taking into consideration the advantages and disadvantages of each technique.

### 1.1. Platelet-Rich Plasma (PRP)

In 1997, Whitman et al. published the first article on the use of platelet-rich plasma (PRP), the first generation of APCs, in oral and maxillofacial surgery. During the preparation of PRP, xenogeneic thrombin and anticoagulant were added. This technique uses exogenous materials and might cause an immunologic and infectious response, making its use controversial [15,18].

PRP plays a vital role in wound healing. The wound-healing process can be divided into three stages: biochemical activation, cellular activation and cellular response. First, there is a conversion of the mechanical injury into biochemical signals. This cascade is triggered by the Hageman factor in the serum. As a result of the disruption of microcirculation, the plasma comes into contact with tissue proteins and the basement membrane, activating the Hageman factor and platelets. The clotting cascade enables fibrin to facilitate homeostasis, and it activates thrombin. Thrombin, calcium chloride and ADP trigger the activation of platelets, leading to the release of alpha granules from platelets, with the subsequent secretion of a large variety of growth and differentiation factors [19].

The complement cascade also includes the release of substances that are important for wound repair. During this process, bradykinin is produced, which causes vasodilatation and the activation of plasminogen to produce plasmin, which degrades the fibrin. The fibrin degradation causes monocyte migration and vasodilatation. The third stage is the cellular response. In this stage, GFs are released from platelets. These GFs signal the local epithelial and mesenchymal cells to migrate, divide and enhance the synthesis of the collagen matrix. The platelet count in PRP is 338% of the platelet count of the whole blood [20]. PRP enhances bone deposition and the quality of bone regeneration during bone augmentation as GFs from autologous blood are delivered to the treatment site [20]. Moreover, platelet and GF concentrations in PRP are, on average, 3‒5 times higher in PRP than in peripheral blood.

### 1.2. The Technique

**Figure 1:**
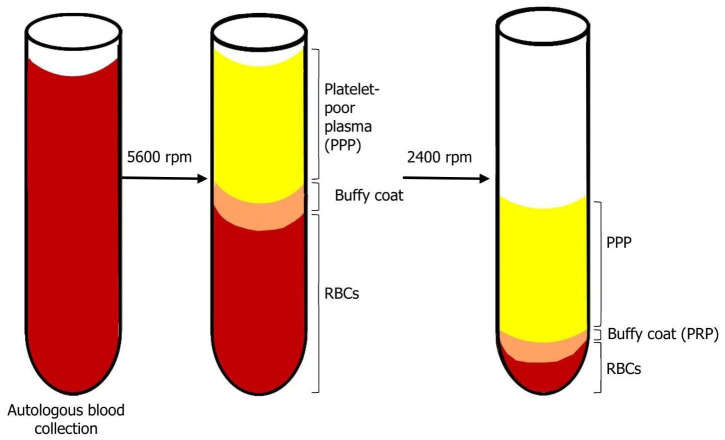
Blood centrifugation after collection. After the first centrifugation period, there is a separation of two layers: on top—platelet-poor plasma (PPP), on bottom—red blood cells (RBCs) and buffy coat. The products of the second centrifugation period are: top—PPP; bottom—buffy coat (PRP) and residual RBCs.

In the pre-operative period, 450 mL blood is collected in a sterile centrifuge tube, containing citrate–phosphate–dextrose solution (as anticoagulant). First, it is centrifugated (Medtronic Electromedic, Elmd-500 Autotransfusion system, Parker, CO, USA) at 5600 rpm. The result of this stage is the separation into two layers: first layer—platelet-poor plasma (PPP); second layer—red blood cells (RBCs) and buffy coat, which contains platelets and white blood cells (WBCs) (see Figure 1). Only the layer of RBCs and buffy coat then continues to the second stage of separation. The second centrifugation period is processed at 2400 rpm in order to separate the buffy coat into PRP and residual RBCs. When the surgeon needs to use the PRP, thrombin is dissolved in 10 mL 10% calcium chloride in a sterile cup. Then, 7 mL PRP and 2 mL air are aspirated into a 10 mL syringe with a 14 gauge catheter. Then, a 1 mL mixture of thrombin + calcium chloride is aspirated into the syringe. Within 5–30 s, the thrombin enables the polymerization of fibrin into a insoluble gel, platelet degranulation and the release of GFs and cytokines. The gel is injected to the desirable site. It should be noted that there is a difference in platelet quantity: platelet and WBC count is higher in younger people and higher in females compared to males [15,21,22].

### 1.3. Platelet-Rich Fibrin (PRF)—The Second Generation of Platelet Concentrates

In 2000, Choukroun et al. reported on a new alternative to PRP: platelet-rich fibrin (PRF) [16,23]. This biomaterial was developed for use in oral and maxillofacial surgical procedures. The application of PRF is different from PRP and does not require use of any anticoagulant or thrombin, only centrifuged autologous blood. Fibrin is an insoluble molecule that is the activated form of fibrinogen, a soluble molecule by thrombin, factor XIII, calcium ions and fibronectin. Fibrin is part of the last stage in the coagulation cascade. The fibrin molecule plays a vital role during coagulation. This molecule is found in platelet alpha granules and in plasma. Fibrin becomes a biological adhesive that enables the stabilization of the initial platelet cluster during coagulation. The fibrin network is the first to reach the injured tissue. The regeneration capacity of PRF is due to its angiogenesis potential, which can be explained by the 3D fibrin matrix that can carry, at the same time, cytokines and GFs such as VEGF, IGF, TGF-β1 and PDGF. As previously described, these factors play vital roles in the regeneration process. Furthermore, during angiogenesis, endothelial cells express αvβ3 integrin, which enables the interaction between the endothelial cells and fibrin, fibronectin and vitronectin. Moreover, PRF can also recruit stem cells from the circulating blood. Chouckroun et al. reported an immunological benefit of PRF. These benefits could explain why, while using this technique, there are fewer post-operation infections [23,24].

### 1.4. The Technique

**Figure 2:**
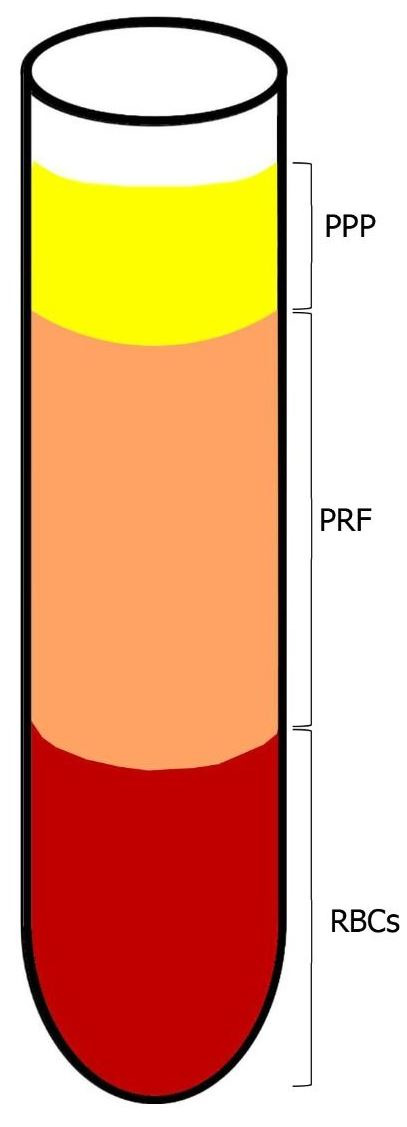
Blood centrifugation after collection. The layers after centrifugation period are: on bottom—RBCs, middle layer—fibrin clot layer (PRF) and on top—PPP.

As described by Chouckroun et al., IV blood is collected in 10 mL tubes with no anti-coagulant addition; it is then centrifugated at 3000 rpm for 10 min. At the end of the procedure, three layers are obtained: 1. Bottom—RBC layer; 2. Middle—Fibrin clot layer (PRF); 3. Top—serum layer (PPP) (see Figure 2). As mentioned above, there is no anticoagulant addition. Hence, the coagulation process starts immediately when the blood comes into contact with the glass tube [16].

### 1.5. Injectable PRF

Miron et al. published a modification to PRF: a liquid formulation of PRF injectable PRF (i-PRF) with no use of anticoagulants. As compared to PRP, after 10 days, i-PRF released higher levels of GFs such as IGF-1, EGF, PDGF-AA/AB. Furthermore, i-PRF induced the highest fibroblast migration, while PRP induced higher levels of cell proliferation [25]. Fujioka-Kobayashi et al. noted that modification to centrifugation speed and time influence GF release. As centrifugation speed decreases, GF and leukocyte release from the PRF clot is increased [26].

### 1.6. Concentrated Growth Factor (CGF): The Newest Platelet Concentrate

In 2006, Sacco [26] reported on the newest platelet concentrate—CGF. CGF is produced in a manner that is similar to that used to produce PRF, but it involves a different centrifuge speed (Medifuge, Silfradent, Italy). CGF contains GFs such as VEGF, PDGF, IGF-I and TGF-β1. Compared to PRF, CGF contains a denser and richer GF‒fibrin matrix. Furthermore, CGF has a 3D fibrin network in which growth factors are closely bound to one another. This provides the slow release of growth factors, which helps with wound healing [18,27].

### 1.7. The Technique

**Figure 3:**
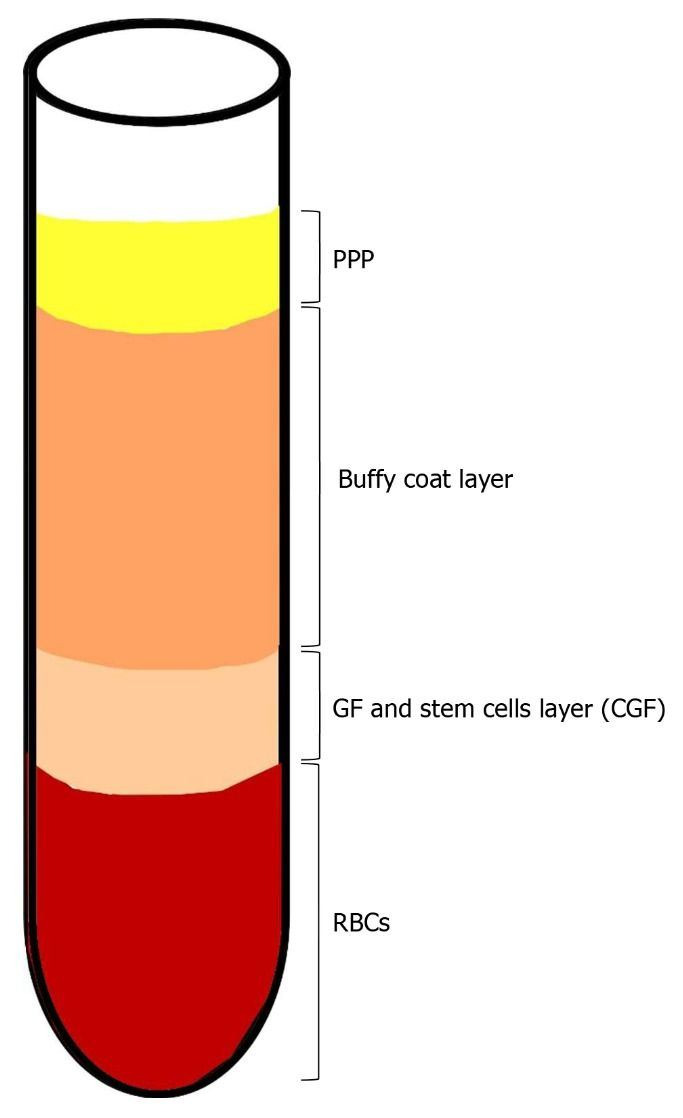
Blood centrifugation after collection. At the end of the centrifugation period, four layers are obtained: 1. Bottom—RBC layer; 2. GF and stem cell layer (CGF); 3. Buffy coat layer; 4. Top—serum layer (PPP).

As described by Bozkurt et al., IV blood is collected in two 10 mL glass-coated plastic tubes with no anticoagulant addition. The tubes are immediately centrifuged (Medifuge, Silfradent, S. Sofia, Italy) in the following manner: 30’’ acceleration, 2’ 2700 rpm, 4’ 2400 rpm, 4’ 2700 rpm, 3’ 3000 rpm and 36’’ deceleration until end. At the end of the procedure, four layers are obtained from bottom to top: RBC layer, GF and stem cell layer (CGF), Buffy coat layer, serum layer (PPP) (see Figure 3). Then, the CGF layer is separated using sterile surgical scissors. The CGF clot is then squeezed in a special box at a thickness of 1 mm. The CGF is then placed over the target site [28].

### 1.8. Periodontal Regeneration

The cementum, gingiva, periodontal ligament (PDL) and alveolar bone serve as the tooth-supporting tissues (periodontium). Periodontitis is a chronic multifactorial inflammatory disease which is primarily characterized by the destruction of alveolar bone and tooth-supporting connective tissue, which is manifested by a loss of clinical attachment, presence of periodontal pocketing and bleeding on probing [29]. Lack of treatment may lead to tooth loss. Scaling and root planning (SRP) is an initial treatment for periodontitis, enabling plaque removal and local inflammation control [30]. These therapies cannot provide reattachment of the periodontium tissues to teeth. The aim of periodontal regeneration is to regenerate the tooth-supporting tissues: to form new bone, cementum and supportive PDL in order to provide optimal structure and function [31]. As previously described by Gottlow et al., periodontal regeneration is based on guided tissue regeneration, which enables selected cell populations to reach the target site together with barriers in order to prevent the migration of epithelial cells to the regenerating site. As a result, PDL cells can migrate and enable connective tissue attachment and regeneration [32] As previously described by Larsson et al., recent advances in this field include the use of diverse biomaterials, GFs, stem cells and bone replacement grafts [33]. The current narrative review will discuss the use of three APC techniques in the field of periodontology: treatment of gingival recession, furcation defects and intrabony defects.

### 1.9. Facial Rejuvenation

In recent years, there has been an increase in the use of the techniques in the field of skin rejuvenation due to the high concentration of growth factors in platelets. APCs are used in facial rejuvenation of the periorbital area and wrinkles, the treatment of acne scars and in lip augmentation. Kim et al. demonstrated that PRP stimulates dermal fibroblast proliferation, collagen synthesis and matrix metalloproteinase expression, thereby aiding facial rejuvenation [34]. There are facial rejuvenation procedures that use combined PRP and fat-grafting procedures to achieve facial volume. Rophael et al. indicated that the angiogenesis benefit of PRP (which contains GFs) may explain the mechanism underlying the fat graft retention, especially during the ischemic period after fat injection [35]. Choukroun and Miron indicated that PRF can be utilized for lip augmentation and to treat both acne scars and wrinkles [36]. Wang et al. reported improved results following CGF injections used to treat periorbital wrinkles, as evaluated at 3 months after treatment [37]. 

This narrative review is designed to provide the clinician with an overview of APC evolution over the past decade. The uses of the different methods will be elaborated, to provide the clinician with reliable and useful information for clinical implementation and to consider novel uses of the three techniques in the fields of periodontal regeneration and facial rejuvenation. To the best of our knowledge, this is the first time in the literature that a narrative review article is devoted to discussing the uses of the three APC methods in the areas of periodontal regeneration and skin rejuvenation, summarizing the indications for using each method, as well as advantages and limitations, and presenting the reader with the most up-to-date information, mostly collected over the last decade.

## 2. Materials and Methods

**Figure 4:**
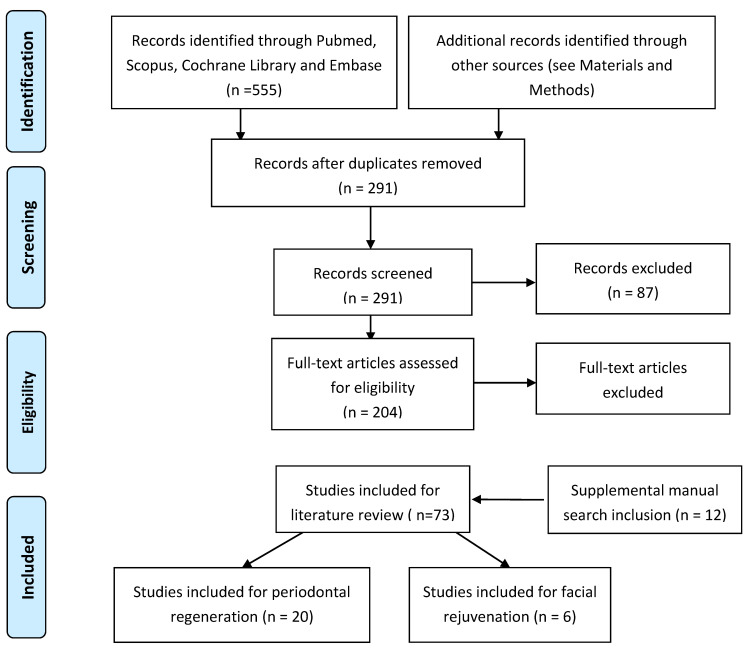
Flowchart of the search strategy.

The Pubmed, Cochrane Library, Scopus and Embase databases were searched from January 1997 to December 2020 to find published studies on the effects of different autologous platelet concentrates on periodontal regeneration and facial rejuvenation. (see Figure 4) The keywords used in the preliminary search were as follows: “VEGF”, “TGF-b1”, “PRP”, “PRF”, “CGF”, AND “periodontal regeneration” or “facial rejuvenation”. The selection included all studies presented in the English language that investigated the effect of autologous platelet concentrates on periodontal regeneration and facial rejuvenation. The review process, including search and selection (identification, screening, eligibility of included studies), was performed according to the PRISMA criteria. In the selection process, all articles were selected by abstract and title; abstracts were initially read by two independent researchers to identify potentially eligible full-text papers. All authors discussed and agreed upon which articles met the inclusion criteria and which articles to exclude. 

The following criteria were applied for APCs in periodontal regeneration: 

Inclusion criteria:

- Study design: randomized controlled trials (RCTs), cohort studies, cross-sectional studies only in the English language;
- Population: only studies on humans, with a minimum sample size of 10 patients and no restriction in terms of patient ages;
- Intervention: regenerative periodontal surgery on interproximal bony defects (IBD) and furcation defects (FD);
- Types of outcome: probing pocket depth recovery (PPDR) and clinical attachment level gain (CALG).

Exclusion criteria:

- Absence of baseline data before periodontal surgery;
- Patients with systemic diseases or craniofacial anomalies;
- No training in oral hygiene;
- Follow-up < 6 months.

The following criteria were applied for APCs in facial rejuvenation: 

Inclusion criteria:

- Study design: randomized controlled trials (RCTs), cohort studies, cross-sectional studies, case reports and case series only in the English language;
- Population: only studies on humans; for CGF, studies on animals were included;
- Intervention: facial skin rejuvenation, facial wrinkles, atrophic acne scars;
- Types of outcome: clinical or histologic evaluation.

Exclusion criteria:

- Hair and nail restoration;
- Patients with alopecia;
- Review articles.

For the manual search, we selected six journals (Journal of Periodontology, Periodontology 2000, Journal of Clinical Periodontology, Annals of Dermatology, Aesthetic Plastic Surgery, Journal of Cosmetic Dermatology). In addition, the references of the selected articles were evaluated to find additional publications by manual searches.

## 3. Results

The aim of the present study was to evaluate the state of the art in the use of autologous platelet concentrates in periodontal regeneration and facial rejuvenation. The electronic search identified 741 references (see Figure 4). Citations that were not connected with the topic were rejected and duplicates eliminated. Titles and abstracts were selected according to the inclusion and exclusion criteria; the articles that presented at least one inclusion criteria and no exclusion criteria in the abstract were kept. After evaluation, 204 records were screened on the basis of titles and abstracts, and full texts were read and analyzed. A manual search was performed to find supplementary articles. Consequently, 12 new titles were considered, and 131 that did not have appropriate full texts were excluded at this stage. In total, 73 articles were included for the literary review on APCs; of these, 20 were included in the review on periodontal regeneration and six studies on facial rejuvenation.

### 3.1. PRP

Seven clinical studies on PRP use for infrabony defects were included in the current review (see Table 1). Some studies found that over a period of 6 months, the addition of PRP to a bovine-derived xenograft (BDX) improved the clinical periodontal response. Hanna et al. demonstrated good clinical results in terms of probing depth and clinical attachment loss, in comparison to the use of a graft alone [38]. The combination of PRP and BDX is effective in infrabony defects as compared to GTR alone [39,40]. However, PRP did not provide clinical benefit with β-TCP in the treatment of infrabony defects in patients with periodontitis [41]. Furthermore, at 12 months, the use of PRP failed to improve the results obtained with BDX [42,43]. Piemontese et al. [44] have shown that the addition of PRP to demineralized freeze-dried bone allografts (DFDBA) is effective in pocket depth (PD) reduction and clinical attachment level (CAL) gain. As previously described by Kobayashi et al., at earlier time points, PRP provides more rapid delivery of growth factors, as compared to PRF [45]. Moreover, the addition of PRP to bone autografts and allografts has been shown to induce dense matured bone with organized trabeculae [18] and the use of PRP increases bone deposition and improves the quality of bone for augmentation of edentulous sites to aid future implant placement [20]. Wroblewski et al. have also demonstrated that PRP facilitates graft placement and stability [19]. However, there are also a few limitations to the use of PRP. Thrombin inhibits cell migration during bone repair [46]. As the source of thrombin is exogenous (i.e., bovine), there may a risk of transmissible infectious diseases, e.g., bovine spongiform encephalopathy [47]. In addition, there is a risk of coagulopathies. Specifically, the use of bovine thrombin increases the risk of the production of antibodies to factors V and XI, which increases the risk of coagulopathies. The exogenous thrombin can react with the patient’s immune system. The cross-reactivity between the anti-bovine factor V antibody and the endogenous factor V may cause factor-V deficiency after the use of thrombin in PRP. Note that the reaction is dependent on the source, purity and quality of the thrombin used [48]. Another limitation is that, when used in sinus augmentation, PRP requires living cells [20].

### 3.2. PRF

Since 2009, the use of the PRP technique has diminished, as demonstrated in Table 1 by the number of studies published since 2009 regarding the use of PRF. Lekovic et al. [49] tested the use of PRF as an adjunct to BDX, and the clinical parameters were significantly improved. Similar results were also reported when PRF was used only with the open-flap debridement (OFD) procedure [50,51,52,53,54]. A statistically significant difference was observed in terms of regeneration in the treatment of furcation defects by Sharma et al. [55]. PRF combined with OFD provided significantly higher GCF concentrations of angiogenic biomarkers and better periodontal healing in terms of conventional flap sites [56]. The addition of PRF did not improve PPD reduction and CAL gain when added to Emdogain placement [57]. The combination of OFD/PRP/hydroxyapatite (HA) improved the outcome in intrabony defects with respect to PRF alone [58].

Pradeep et al. [59] and Bajaj et al. [60] studied the effects of PRP and PRF in infrabony and furcation defects, respectively. In the first study, similar PD reduction and CAL gain were observed in infrabony defects treated with PRP or PRF, and both of them showed a significant improvement compared to open-flap debridement alone. PRF was slightly more effective than PRP in the treatment of furcation defects. Because PRF is less time-consuming and less technique-sensitive, it may represent a better treatment option than PRP [59]. As described in Table 2, the use of PRF presents a number of advantages: compared to PRP, PRF provides advantages in terms of the relative amount and diversity of cytokines and their release over time [25,61]. As previously described by He et al., the use of PRF is associated with the steady release of GFs over 10 days [61]. As compared to PRP, the use of PRF does not require any anticoagulant, thrombin or blood manipulation. Therefore, this approach provides immunological biocompatibility [25]. The material is easy to prepare and the technique is simple to use. Another advantage is the reduction of patient discomfort during the early stages of wound healing [24]. As compared to PRP, the time and cost of preparation are lower in the PRF technique. However, there are also a few limitations to the use of PRF: Miron et al. noted that, while there have been studies to support the use of PRF for periodontal purposes and soft tissue repair, we still lack sufficient data regarding the effects of PRF on hard tissue repair during bone regeneration [24]. Another limitation is that, since PRF is prepared from autologous blood, only small quantities can be produced at one time and, in order to obtain usable PRF, the preparation process must be quickly completed.

### 3.3. CGF

Regarding CGF, there are limited studies available with relation to periodontal regeneration. Only one study reported the effect of CGF in infrabony defects. The authors concluded that CGF can enhance bone regeneration and reduce the depth of periodontal intrabony defects. When combined with a xenograft, CGF might be a superior scaffolding material [62]. CGF presents number of advantages. The use of CGF involves a simple and inexpensive procedure [63]. As mentioned above regarding the use of PRF, the use of CGF requires no exogenous additions, such as thrombin or bovine calcium chloride. Therefore, the probability of cross-contamination is low. As mentioned above regarding PRF, the use of CGF is associated with the steady release of growth factors over 7–10 days. There are also some limitations to the use of CGF: for example, the platelet count in CGF is influenced by the blood pH [63]. Moreover, changes in the blood pH may disturb cell proliferation. Moreover, the duration of CGF preparation and the blood volume may influence the results.

### 3.4. Facial Rejuvenation

As mentioned in Table 3, PRP enhances rejuvenation by inducing collagen synthesis and dermal fibroblast proliferation. Facial aging is caused by extracellular matrix (ECM) alterations and poor fibroblast proliferation. PRP is a more natural treatment for skin rejuvenation and is also used to treat acne scarring and alopecia. There is a specific need for long-term, follow-up studies to evaluate whether the benefits of PRP persist over time. PRP application (even as a single application) could be considered as an effective and safe procedure for the rejuvenation of facial skin. There is a need for further research to clarify the specific biological and clinical effects of this procedure [64,65,66]. Further studies will be needed to determine if platelet concentrates are a valid aid in dermatology and if they can be considered as an alternative to or support for other therapies.

## 4. Discussion

The current narrative review includes RCT studies, clinical studies, cross-sectional studies, case reports and case series. The aim was to provide clinicians with up-to-date information about the use of PRP, PRF and CGF in dentistry, including the clinical indications, advantages and limitations of each technique. This article has a clinical orientation and was written in a manner designed to provide clinicians with reliable and useful information applicable to their work and interest in novel uses of the three techniques in the field of periodontal regeneration and facial rejuvenation.

### 4.1. Periodontal Regeneration

Periodontal regeneration, following destructive episodes of periodontal disease, must involve not only the affected alveolar bone but also the periodontal ligament and the root cementum [68].

The three techniques described in this article differ in GF amounts [2]. Mazuki et al. found that PRF and CGF preparations contained significant amounts of growth factors as compared to PRP. Moreover, these two preparations are more capable of inducing angiogenesis and, as a result, increase the wound-healing regeneration as compared to PRP [69]. The three techniques are also different in the use of thrombin and calcium chloride (PRP) or non-use of them and in the complexity of the preparation protocol—PRP preparation requires two stages of centrifugation, while PRF and CGF are simpler and require only one stage.

As previously described in Table 1, Hanna et al. demonstrated good clinical results in terms of probing depth and clinical attachment loss, in comparison to the use of a graft alone [38]. The combination of PRP and BDX is effective in infrabony defects as compared to GTR alone [39,40]. Furthermore, at 12 months, the use of PRP failed to improve the results obtained with BDX [42,43]. These results might be affected by the different follow-up periods or due to differences in the study design (split-mouth vs. parallel design).

Since 2009, the use of the PRP technique has diminished, as demonstrated in Table 1 by the number of studies published since 2009 regarding the use of PRF. Lekovic et al. tested the use of PRF as an adjunct to BDX, and the clinical parameters were significantly improved [49]. Similar results were also reported when PRF was used only with the open-flap debridement (OFD) procedure [50,51,52,53,54]. A statistically significant difference was observed in terms of regeneration in the treatment of furcation defects by Sharma et al. [55]. Pradeep et al. and Bajaj et al. studied the effects of PRP and PRF in infrabony and furcation defects, respectively [59,60]. In the first study, similar PD reduction and CAL gain were observed in infrabony defects treated with PRP or PRF, and both of them showed a significant improvement compared to open-flap debridement alone. PRF was slightly more effective than PRP in the treatment of furcation defects.

Regarding CGF, there are limited studies available in relation to periodontal regeneration. Its use has been proposed in dentistry for various situations, ranging from the filling of extraction sockets [70] to the filling of a cavity after cystectomy [71], sinus augmentation procedures [63,72,73], simple GBR procedures [74,75] or as a membrane support in recession coverage [28,76,77,78]. It was demonstrated to possess the capacity to accelerate new bone formation [79]. Moreover, it can be used alone or with autologous bone particles or biomaterials [75,80]. Bozkurt et al. reported that using CGF, together with coronally advanced flap (CAF), to treat maxillary gingival recession led to significantly wider keratinized gingiva and thicker gingiva, as compared to CAF, 6 months after the procedure [28]. No significant differences between the treatments were noted for recession depth, complete root coverage or mean root coverage. Connective tissue graft (CTG) is superior to CGF for enhancing keratinized tissue thickness and width during surgical root coverage procedures [76]. CGF is widely used in implant surgery. The use of CGF led to increased implant stability and osteo-integration [81,82] and may be preferable due to decreased postoperative pain [82]. In a 12-month RCT, Isler et al. compared the use of a collagen membrane with the use of CGF in the regenerative surgical treatment of peri-implantitis. They found that both approaches yielded significant improvements in both clinical and radiograph assessments [83]. The use of sticky bone in maxillary sinus augmentation procedures has provided new bone formation and predictable clinical results. It was also evident in cone beam computer tomography (CBCT) [73]. Only one study reported the effect of CGF in infrabony defects. The authors concluded that CGF can enhance bone regeneration and reduce the depth of periodontal intrabony defects. When combined with a xenograft, CGF might be a superior scaffolding material [62].

### 4.2. Facial Rejuvenation

In recent years, there has been increased use of the techniques presented in the field of facial rejuvenation. The added value of using these techniques is the ability to deliver a high amount of GF to the scar, acne, periorbital fine line and pigmentation target areas. The alternatives for facial rejuvenation are hyaluronic acid injections and fat grafting. The former is expensive and its effects last only up to 12 months, and the latter can cause unpredictable swelling. The periorbital region is an area of the face that enables us to estimate a person’s age and has aesthetic and beauty aspects. This area is prone to pigmentation, wrinkles, erythema, xerosis, decreased skin elasticity and melanosis, all of which corelate to the person’s age. PRP enhances rejuvenation by inducing collagen synthesis and dermal fibroblast proliferation. Facial aging is caused by extracellular matrix (ECM) alterations and poor fibroblast proliferation.

Samadi et al. published a review regarding the multiple clinical uses of PRP for aesthetic and regenerative medicine [84]. Mayes et al. sought to identify the most influential parameters for assessing a woman’s age and found that wrinkles and hyperpigmentation have the strongest relationships with perceived age [85]. PRP is a more natural treatment for skin rejuvenation and is also used to treat acne scarring and alopecia. Kim et al. demonstrated that PRP stimulates dermal fibroblast proliferation and collagen synthesis [34].

There are facial rejuvenation procedures that combine PRP and fat grafting to achieve facial volume. Rophael et al. indicated that the angiogenesis benefit from PRP may explain the mechanism underlying fat graft retention, especially during the ischemia period after fat injection [35]. Cameli et al. examined facial skin rejuvenation in 12 patients after three sessions of PRP injections (delivered at 1-month intervals). Their evaluation revealed improvements in skin texture, elasticity and smoothness [64]. The authors concluded that further investigation of this procedure is needed. There is a specific need for long-term, follow-up studies to evaluate whether the benefits of PRP persist over time. PRP application (even a single application) could be considered as an effective and safe procedure for the rejuvenation of facial skin. Liquid injectable PRF can be used instead of PRP for facial rejuvenation. As previously described, PRF does not require the addition of anticoagulants and therefore will not inhibit tissue regeneration. As demonstrated by Choukroun and Miron, PRF can be used for lip augmentation, as well as for the treatment of acne scars and wrinkles [36]. Sclafani et al. examined the efficacy of a single injection of autologous platelet-rich fibrin matrix for the correction of nasolabial fold defects [66]. The results were examined in terms of a wrinkle assessment score and a global aesthetic improvement scale. Digital photographs were taken before and after the procedure. Follow-up evaluations were conducted at 1, 2, 6 and 12 weeks after treatment. The researchers found that treatment with PRF yielded positive results, as compared to other dermal stimulators. The effects appeared quickly and the technique was easy to use. There is a need for further research to clarify the specific biological and clinical effects of this procedure [66]. The regenerative efficacy of CGF is due to the release of GF over a long period of time and its bioactivity advantage. Hu et al. examined the effect of CGF on periorbital wrinkles. They reported on the use of CGF injections to treat periorbital wrinkles. CGF was added to an adipose graft. The results of this treatment were examined in terms of wrinkles, volume and complications at 3 to 6 months after the injection of CGF. Significant results were obtained using CGF [37]. The main studies on facial rejuvenation are reported in Table 3. In addition to their dental indications, APCs may be used as treatment modalities in different medical indications, such as autoimmune diseases, oral lichen planus [86] and alopecia areata [87]. Further studies will be needed to determine whether platelet concentrates are a valid aid in dermatology and if they can be considered as an alternative or support to other therapies.

## 5. Conclusions

This narrative review is designed to provide clinical information regarding the use of three APC techniques in periodontal regeneration and facial rejuvenation: PRP, PRF and CGF. The advantage of the APCs is the fact that a large amount of GF can be delivered to the target site and encourage angiogenesis and wound healing. PRP is the most established among the techniques reviewed in this article and is used in soft and hard tissue repair. At early time points, PRP provides more rapid delivery of GFs to the target site as compared to PRF and CGF. As a result of the addition of PRP to a bone autograft, dense and mature bone is formed. However, the transmission of infectious diseases and coagulopathies serves as a crucial limitation to the PRP technique and should be taken into consideration. In contrast to PRP, PRF and CGF require only centrifuged autologous blood and therefore provide immunological biocompatibility. PRF is effective mostly for soft tissue repair such as gingival recession coverage and furcation defects and is also used for intrabony defects. CGF is used in oral surgery, mostly for hard tissue regeneration. This narrative review also discussed the use of the three APC techniques in facial rejuvenation. The added value of using these techniques is the ability to deliver a high number of GFs to the target site. The use of PRP promotes tissue remodeling in aged skin and may be used as an adjuvant treatment to lasers. PRF holds significant potential for stimulated dermal augmentation. CGF treatment could improve the survival and quality of fat grafts. As this is a narrative review, this serves as a limitation of this study. Thus, interpretation should be made with caution. Future high-quality studies, e.g., randomized controlled trials, should be conducted; future systematic reviews and meta-analyses regarding the current review topic are also warranted.

## Figures and Tables

**Table 1:** Comparison between the 3 APC techniques, PRP, PRF and CGF, in periodontal regeneration.

First Author	Participants	Methods	Treatment	Parameters	Follow-Up (Months)	Results (mm)	Author’s Conclusions
PRP							
Camargo et al. [39]	18 split-mouth design	5600 rpm/10% trisodium citrate solution	IBD	PPDR	6	GTR 3.58 ± 0.94 PRP/BDX/GTR 4.95 ± 0.85	PRP and BDX provide an added regenerative effect to GTR in promoting the clinical resolution of intrabony defects in patients with severe periodontitis.
				CALG		GTR 2.55 ± 1.22 PRP/BDX/GTR 4.34 ± 1.32	
Lekovic et al. [40]	21 split-mouth design	5600 rpm/10% trisodium citrate solution	IBD	PPDR	6	PRP/BDX 3.96 ± 0.98 PRP/BDX/GTR 4.2 ± 0.90	Combinations of PRP/BPBM/GTR and PRP/BPBM are effective in the treatment of intrabony defects present in patients with advanced chronic periodontitis. The GTR adds no clinical benefit to PRP/BPBM.
				CALG		PRP/BDX 3.81 ± 0.74 PRP/BDX/GTR 4.14 ± 0.81	
Hanna et al. [38]	13 RCT, split-mouth design	SmartPReP, 10% CaCl2 mixed with 1000 U.S. units of topical thrombin	IBD	PPDR	6	BDX 2.53 PRP/BDX 3.54	The addition of PRP to BDX for the treatment of intabony defects demonstrated good clinical results with respect to the use of graft alone.
				CALG		BDX 2.31 PRP/BDX 3.15	
Dori et al. [42]	30 Parallel design study	Curasan PRP kit 2400 rpm/10 min-3600 rpm for 15 min	IBD	PPDR	12	BDX/GTR 5.5 ± 2.4 PRP/BDX/GTR 5.5 ± 2.2	Regenerative surgery with both PRP/BDX/GTR and BDX/GTR, significant PD reductions and CAL gains were found; the use of PRP failed to improve the results obtained with BDX/GTR.
				CALG		BDX/GTR 4.6 ± 2.4 PRP/BDX/GTR 4.5 ± 2.0	
Piemontese et al. [44]	60 RCT, double-masked	SmartPReP, 10% CaCl2 mixed with 1000 U.S. units of topical thrombin	IBD	PPDR	12	DFDBA 2.6 ± 2.2 DFDBA/PRP 4.3 ± 1.7	The combination of PRP and DFDBA led to a significantly greater clinical improvement in intrabony defects compared to DFDBA with saline. No statistically significant differences were observed in the hard tissue response between the two groups.
				CALG		DFDBA 2.3 ± 2.4 DFDBA/PRP 3.5 ± 2.1	
Harnack et al. [41]	22 Prospective RCT, split-mouth	Curasan PRP kit-2400 rpm/10 min-3600 rpm for 15 min	IBD	PPDR	6	HA 0.28 HA/PRP 0.8	PRP did not improve the results achieved with β-TCP alone in the treatment.
				CALG		HA 0.13 HA/PRP 0.4	
Dori et al. [43]	30 parallel-arm design RCT	Curasan PRP kit-2400 rpm/10 min-3600 rpm for 15 min	IBD	PPDR	12	BDX 5.3 ± 1.7 BDX/PRP 5.2 ± 1.6	The use of PRP failed to improve the results obtained with BDX alone.
				CALG		BDX 4.6 ± 1.6 BDX/PRP 4.6 ± 1.7	
PRF							
Pradeep et al. [50]	20 Prospective RCT, split-mouth	Su et al.	FD	PPDR	6	OFD 0.8 ± 1.31 OFD/PRF 2.3 ± 1.41	A statistically significant difference was observed in all the clinical and radiographic parameters at the sites treated with PRP as compared with those with OFD. However, all the furcation defects retained their degree II status.
				CALG		OFD 0.1 ± 1.10 OFD/PRF 2.5 ± 1.64	
Sharma et al. [55]	18 Prospective RCT, split-mouth	Choukroun et al. 3000 rpm 10 min	FD	PPDR	9	OFD 2.89 ± 0.68 OFD/PRF 4.06 ± 0.42	Significant improvement with autologous PRF implies its role as a regenerative material in the treatment of furcation defects.
				CALG		OFD 1.28 ± 0.46 OFD/PRF 2.33 ± 0.49	
Sharma et al. [51]	35 (56 sites) longitudinal interventional study	Choukroun et al. 3000 rpm 10 min	IBD	PPDR	9	OFD 3.21 ± 1.64 OFD/PRF 4.55 ± 1.87	There was greater PPD reduction, CAL gain and bone fill at sites treated with PRF with conventional open-flap debridement compared to conventional open-flap debridement alone.
				CALG		OFD 2.77 ± 1.44 OFD/PRF 3.31 ± 1.76	
Thorat et al. [52]	32 CCT	Choukroun et al. 400 g 12 min	IBD	PPDR	9	OFD 3.56 ± 1.09 OFD/PRF 4.69 ± 1.45	There was greater reduction in PD, more CAL gain and greater intrabony defect fill at sites treated with PRF than the open-flap debridement alone.
				CALG		OFD 2.13 ± 1.71 OFD/PRF 4.13 ± 1.63	
Lekovic et al. [49]	17 Prospective RCT, split-mouth	1000 g 10 min	IBD	PPDR	6	PRF 3.29 ± 0.70 PRF/BDX 4.38 ± 0.80	PRF can improve clinical parameters associated with human intrabony periodontal defects, and BDX has the ability to augment the effects of PRF in reducing pocket depth, improving clinical attachment levels and promoting defect fill.
				CALG		PRF 2.18 ± 0.71 PRF/BDX 3.76 ± 0.77	
Aydemir et al. [57]	28 RCT, split-mouth	400 g 10 min	IBD	PPDR	6	EMD 3.88 ± 1.26 PRF/EMD 4.00 ± 1.38	Both therapies resulted in significant clinical improvement in IBD treatment. Addition of PRF did not improve the clinical and radiographic outcomes.
				CALG		EMD 3.29 ± 1.30 PRF/EMD 3.42 ± 1.28	
Arabaci et al. [56]	26 RCT, split-mouth design	2800 rpm Tunalı et al.	IBD	PPDR	9	OFD 2.84 ± 0.97 OFD/PRF 3.54 ± 1.11	PRF membrane combined with OFD provides significantly higher GCF concentrations of angiogenic biomarkers for 2 to 4 weeks and better periodontal healing in terms of conventional flap sites.
				CALG		OFD 2.22 ± 0.75 OFD + PRF 2.88 ± 1.03	
Patel et al. [53]	26 RCT, split-mouth design	Choukroun et al. 3000 rpm 10 min	IBD	PPDR	12	OFD 2.40 ± 0.84 OFD/PRF 3.80 ± 1.48	The adjunctive use of PRF with conventional OFD may be usefully used in the treatment of intrabony defects.
				CALG		OFD 2.10 ± 0.74 OFD + PRF 3.70 ± 0.67	
Pradeep et al. [58]	57 (90) RCT	Choukroun et al. 3000 rpm 10 min	IBD	PPDR	9	OFD 2.97 ± 0.93 OFD/PRF 3.90 ± 1.09 OFD/PRF/HA 4.27 ± 0.98	Treatment of IBD with PRF results in significant improvements in clinical parameters compared to baseline. When added to PRF, HA increases the regenerative effects observed with PRF in the treatment of 3-wall IBDs.
				CALG		OFD 2.67 ± 1.09 OFD/PRF 3.03 ± 1.16 OFD/PRF/HA 3.67 ± 1.03	
Bajaj et al. [54]	17 (44 sites) RCT	Choukroun et al. 3000 rpm 10 min	IBD	PPDR	9	OFD 2.14 ± 1.26 OFD/PRF 3.14 ± 1.26	There is greater bone fill at sites treated with PRF with conventional OFD than conventional OFD alone.
				CALG		OFD 1.59 ± 1.01 OFD/PRF 2.66 ± 1.07	
PRP vs. PRF							
Pradeep et al. [59]	50 (90 sites) RCT	PRP: 5600 rpm/10% trisodium citrate solution PRF:3000 rpm 10 min	IBD	PPDR	6	OFD 2.97 ± 0.93 OFD + PRP 3.77 ± 1.03 OFD + PRF 3.77 ± 1.19	Within the limits of the present study, there was similar PD reduction, CAL gain and bone fill at sites treated with PRF or PRP with conventional open-flap debridement. Because PRF is less time-consuming and less technique-sensitive, it may seem a better treatment option than PRP.
				CALG		OFD 2.83 ± 0.91 OFD + PRP 2.93 ± 1.08 OFD + PRF 3.17 ± 1.29	
Bajaj et al. [60]	37 (72 sites) RCT	PRP: 5600 rpm/10% trisodium citrate solution PRF: Choukroun et al. 400 g 12 min	FD	PPDR	9	OFD 1.58 ± 1.02 OFD + PRP 3.92 ± 0.93 OFD + PRF 4.29 ± 1.04	All clinical and radiographic parameters showed statistically significant improvement at both the test sites (PRF with OFD and PRP with OFD) compared to those with OFD alone. The use of autologous PRF or PRP was effective in the treatment of furcation defects with uneventful healing of sites.
				CALG		OFD 1.37 ± 0.58 OFD + PRP 2.71 ± 1.04 OFD + PRF 2.87 ± 0.85	
CGF							
Xu et al. [62]	54 (120 sites) RCT	Acceleration for 30 s, 2700 rpm for 2 min, 2400 rpm for 4 min, 2700 rpm for 4 min, 3000 rpm for 3 min, deceleration for 36 s	IBD	PPDR		OFD 1.55 ± 0.93 OFD/CGF 2.45 ± 0.76 OFD/BDX 3.72 ± 0.90 OFD/BDX/CGF 4.36 ± 1.03	CGF reduced periodontal intrabony defect depth and, when mixed with Bio-Oss, CGF showed better results in the early period and the effect was more stable.
				CALG		OFD 2.36 ± 0.92 OFD/CGF 3.09 ± 1.14 OFD/BDX 4.18 ± 1.08 OFD/BDX/CGF 4.45 ± 1.13	

Note: Interproximal bony defects (IBD), buccal degree II furcation defects (FD), guided tissue regeneration (GTR), open-flap debridement (OFD), probing pocket depth reduction (PPDR), clinical attachment level gain (CALG), ABG (autologous bone graft), freeze-dried bone allograft (FDBA), bovine-derived xenograft (BDX), β-tricalciumphosphate (b-TCP), hydroxyapatite (HA), EMD (Emdogain). Randomized clinical trial (RCT). Controlled clinical trial (CCT).

**Table 2:** Summary of the the 3 APC techniques, PRP, PRF and CGF—clinical applications, advantages and limitations.

Clinical Applications	Advantages	Limitations
PRP		
1. Treatment of periodontal intrabony defects.	1. Rapid delivery of GFs at earlier time points.	1. Thrombin inhibits cell migration during bone repair.
2. Maxillary sinus augmentation procedure.	2. Adding PRP to autografts and xenografts induced organized bone trabecules.	2. Risk of coagulopathies.
3. Treatment of grade II periodontal furcation defects.	3. PRP facilitates graft placement and stability.	
4. Facial rejuvenation.	4. Improving quality of bone for augmentation of edentulous site for future implant placement.	
PRF		
1. Treatment of intrabony defects.	1. Releasing diversity of cytokines over time as compared to PRP.	1. Lack of sufficient data regarding the effect of PRF on hard tissue repair.
2. Sinus augmentation procedure with simultaneous implant placement.	2. Steady release of GFs over 10 days.	2. Small quantities can be produced at one time.
3. Treatment of grade II periodontal furcation defects.	3. Biocompatibility—the technique does not require any anticoagulants.	3. In order to obtain usable PRF, the preparation process must be quickly completed.
4. Coverage of gingival recession.	4. Easy to prepare and use.	
5. Peri-implant regeneration procedures.	5. Reduce patients’ discomfort during the early stages of wound healing.	
6. Facial rejuvenation.	6. Simple protocol as compared to PRP.	
CGF		
1. Surgical correction of periodontal defects.	1. A simple procedure.	1. The platelet count in CGF is influenced by blood pH. Changes in blood pH may disturb cell proliferation.
2. Coverage of gingival recession.	2. Biocompatibility—the technique does not require any anticoagulants.	The duration of CGF preparation and the blood volume may influence the results.
3. Peri-implant regeneration procedures.	3. Steady release of GFs over 7–10 days.	
4. Facial rejuvenation.	4. The use of CGF is inexpensive.	

**Table 3:** Comparison between the 3 APC techniques, PRP, PRF and CGF, in facial rejuvenation.

First Author	Objective	Methods	Results	Author’s Conclusions
PRP				
Kim et al. [34]	The effects of activated platelet-rich plasma (aPRP) and activated platelet-poor plasma (aPPP) on the remodeling of the extracellular matrix.	Platelet-rich plasma (PRP) and platelet-poor plasma (PPP) were prepared using a double-spin method and then activated with thrombin and calcium chloride.	Platelet numbers in PRP increased 9.4-fold over baseline values. aPRP and aPPP both stimulated cell proliferation, with peak proliferation occurring in cells grown in 5% aPRP. Levels of PIP were highest in cells grown in the presence of 5% aPRP. Additionally, aPRP and aPPP increased the expression of type I collagen, MMP-1 protein and mRNA in human dermal fibroblasts.	aPRP and aPPP promote tissue remodeling in aged skin and may be used as adjuvant treatment to lasers.
Cameli et al. [64]	To evaluate the efficacy and safety of autologous pure PRP dermal injections on facial skin rejuvenation.	Twelve patients underwent 3 sessions of PRP injection at 1-month intervals. The clinical and instrumental outcomes were evaluated before (T0) and 1 month (T1) after the end of treatment by means of transepidermal water loss, corneometry, Cutometer, Visioscan and Visioface. A flow cytometry characterization of PRP and peripheral blood (PB) samples was performed.	Clinical and patient evaluation showed improvement in skin texture. Skin gross elasticity, skin smoothness parameters, skin barrier function and capacitance were significantly improved.	PRP poor in leukocytes can provide objective improvements in skin biostimulation. Although a pilot study, it may be helpful for future investigations on PRP cellularity.
Gawdat et al. [65]	To compare the efficacy and safety of two administration modes of autologous PRP: intradermal injection (ID) and topical application after FCL with that of FCL alone in the treatment of atrophic acne scars.	Thirty patients divided into two groups. Both underwent split-face therapy. Group 1 was administered FCL followed by ID PRP on one side and FCL followed by ID saline on the other. In group 2, one cheek was treated with FCL followed by ID PRP, and the other received FCL followed by topical PRP. Each patient received 3 monthly sessions. The final assessment took place at 6 months.	Combined PRP- and FCL-treated areas had a significantly better response (*p* = 0.03), fewer side effects and shorter downtime (*p* = 0.02) than FCL-treated areas, but there were no significant differences in ID- and topical PRP-treated areas in degree of response and downtime (*p* = 0.10); topically treated areas had significantly lower pain scores.	The combination of topical PRP and FCL as an effective, safe modality in the treatment of atrophic acne scars with shorter downtime than FCL alone and better tolerability than FCL combined with ID PRP.
PRF				
Sclafani et al. [66]	The efficacy of a single injection of autologous platelet-rich fibrin matrix (PRFM) for the correction of deep nasolabial folds (NLFs).	Fifteen adults using a proprietary system (Selphyl; Aesthetic Factors, Inc., Wayne, NJ, USA). Treatment was injected into the dermis and immediate subdermis below the NLFs. Subjects were photographed before and after treatment; NLFs were rated by the treating physician before and after treatment using the Wrinkle Assessment Scale (WAS) and patients rated their appearance at each post-treatment visit using the Global Aesthetic Improvement Scale. Patients were evaluated at 1, 2, 6 and 12 weeks after treatment.	All patients were treated to maximal correction, with a mean reduction in WAS score of 2.12+/−0.56. At 1 week after treatment, this difference was 0.65+/−0.68 but rose to 0.97+/−0.75, 1.08+/−0.59 and 1.13+/−0.72 at 2, 6 and 12 weeks after treatment, respectively (*p* < 0.001). No patient noted any fibrosis, irregularity, hardness, restricted movement or lumpiness.	PRFM can provide significant long-term diminution of deep NLFs without the use of foreign materials. PRFM holds significant potential for stimulated dermal augmentation.
Hassan et al. [67]	This single-center, prospective, uncontrolled study evaluated the efficacy of injectable platelet-rich fibrin (i-PRF) for facial skin rejuvenation using an objective skin analysis system and validated, patient-reported outcome measures.	PRF^®^ PROCESS system technology was used to prepare i-PRP. Eleven female individuals in the study and over 3 months received monthly intradermal injections of i-PRF in 3 facial regions: malar areas (1 mL each side), nasolabial fold (0.5 mL each side) and upper lip skin above the vermilion border (1 mL). The efficacy of the procedures was assessed by objective skin analysis (VISIA^®^) and a subjective patient-reported outcome (FACE-Q) assessment at baseline and after 3 months.	Improvement in skin surface spots (*p* = 0.01) and pores (*p* = 0.03) was seen at 3-month follow-up. Skin texture, wrinkles, ultraviolet spots and porphyrins showed a numerical improvement. FACE-Q scales that measure satisfaction with appearance all showed a significant improvement from baseline, including satisfaction with skin (*p* = 0.002), satisfaction with facial appearance (*p* = 0.025), satisfaction with cheeks (*p* = 0.001), satisfaction with lower face and jawline (*p* = 0.002) and satisfaction with lips (*p* = 0.04). No major adverse effects were reported.	A series of three i-PRF injections resulted in significant rejuvenation of the face skin at 3-month follow-up, as shown by improved skin analysis parameters and patient self-assessment scores.
CGF vs. PRP vs. PRF				
Hu et al. [37]	The impact of the new technique, CGF, on fat graft survival, which was compared with platelet-rich plasma (PRP) and platelet-rich fibrin (PRF).	Nude mice receiving fat graft were divided into PRP group, PRF group, CGF group and saline. The grafts were volumetrically and histologically evaluated at 4, 8 and 12 weeks after fat grafting. In vitro growth factor levels in PRP, PRF and CGF were compared using enzyme-linked immunoassay method. Cell count and real-time polymerase chain reaction were used to evaluate the impact of CGF in medium on human adipose-derived stem cell (hADSC) proliferation and vascular differentiation, respectively.	Fat graft weight was significantly higher in the CGF group than those in the other groups, and histologic evaluation revealed greater vascularity, fewer cysts and less fibrosis. Adding CGF to the medium maximally promoted hADSC proliferation and expression of vascular endothelial growth factor and PECAM-1.	CGF treatment improved the survival and quality of fat grafts.

## Data Availability

Not applicable.
